# Effect of Digestate Modified with Amendments on Soil Health and Plant Biomass under Varying Experimental Durations

**DOI:** 10.3390/ma16031027

**Published:** 2023-01-23

**Authors:** Jiri Holatko, Martin Brtnicky, Adnan Mustafa, Antonin Kintl, Petr Skarpa, Pavel Ryant, Tivadar Baltazar, Ondrej Malicek, Oldrich Latal, Tereza Hammerschmiedt

**Affiliations:** 1 Department of Agrochemistry, Soil Science, Microbiology and Plant Nutrition, Faculty of AgriSciences, Mendel University in Brno, Zemedelska 1, 613 00 Brno, Czech Republic; 2 Agrovyzkum Rapotin Ltd., Vyzkumniku 267, 788 13 Rapotin, Czech Republic; 3 Institute of Chemistry and Technology of Environmental Protection, Faculty of Chemistry, Brno University of Technology, Purkynova 118, 612 00 Brno, Czech Republic; 4 Institute for Environmental Studies, Faculty of Science, Charles University in Prague, Benatska 2, 128 00 Praha, Czech Republic; 5 Agricultural Research Ltd., Zahradni 400/1, 664 41 Troubsko, Czech Republic

**Keywords:** soil enzymatic activity, soil macronutrients, biochar, elemental sulphur

## Abstract

A digestate with amendments provides plants with available nutrients and improves the microbiological properties of treated soil. Modification of a digestate through the addition of a biochar and sulphur source is less well-known. This pot experiment aimed at comparing the short- and long-time fertilization effects of a digestate enriched with biochar, with elemental sulphur, or with a combination of both on soil health and plant biomass. The experiment was carried out with maize, cultivated twice (1st–12th week = pre-cultivation; re-sowing after shoot harvest, 13th–24th = main cultivation) in soil amended with prepared digestate. The digestate used in pre-cultivation was incubated untreated (D) and was then treated with biochar (D + B), with elemental sulphur at a low (LS) and high (HS) dose, or with a combination of both (D + B + LS and D + B + HS). An additional unamended digestate (D) was added to each soil variant before the main cultivation. The application of digestate with a high dose of elemental sulphur and biochar mediated the most significant differences in the soil. The increase (compared to the unamended soil) was of short-term type (+11% and +6% increased total nitrogen and carbon after 12 weeks), then of long-term type (+54% and +30% increased sulphur and arylsulfatase activity after 24 weeks), and later emerged in the 13th to the 24th week of the experiment (+57% and +32% non-inhibited urease, increased N-acetyl-β-D-glucosaminidase and phosphatase). No significant differences in the effect of the applied amendments on dry aboveground plant biomass were observed.

## 1. Introduction

The generation of energy from the cultivation of bioenergy crops can play an important role in replacing fossil fuels with renewable resources [1]. Biogas, as a renewable energy source derived from anaerobic digestion [2], has significant potential for reducing global warming and climate change [3]. Anaerobic digestion provides an alternative option for recycling biodegradable wastes that would otherwise be landfilled or incinerated [4], and can indirectly reduce methane and CO_2_ emissions from landfills [5]. In addition, it promotes nutrient cycling through the production of a nutrient-rich end product, digestate [6,7]. The application of digestate as a fertilizer returns most of the nutrients necessary for plant growth back to the arable soil [6,8,9] while maintaining soil fertility [10], improving soil structure, and replenishing soil organic matter [11,12]. Digestate provides an alternative to energy-intensive mineral fertilizer production [13,14,15] and can replace mineral fertilizers as a source of readily available nitrogen, phosphate, and potassium [16]. Moreover, by adding macro- and micronutrients to the soil, it can effectively promote plant growth. The organic and biological part of the digestate can alter the turnover of soil organic matter (SOM) and change the physico-chemical and microbiological properties of the soil [17]. Digestate also positively influences the activity of soil microorganisms [18], which are involved in nutrient cycling, SOM mineralization, and humus and soil structure formation.

It has also been suggested that digestate contributes to soil organic carbon (SOC) formation and its dynamics [19,20], because digested external organic matter (EOM) has higher stability in soil than undigested EOM [21,22,23]. Positive effects of digestate application on soil nutrient status and their potential loss through leaching have been the focus of many recent studies [24]. Nitrogen in the digestate is predominantly in ammonium form and is susceptible to loss by leaching and volatilization following its conversion to either nitrate or ammonia [25]. In addition to concerns about environmental damage due to the eutrophication of surface water sources, nitrogen losses through ammonia emissions also reduce fertilizer efficiency and the amount of nutrients used by field crops [26].

However, co-application of digestate with other amendments rich in recalcitrant carbon such as biochar [27,28,29,30] may improve soil nutrients and stability and prevent losses due to leaching or rapid transformation. Biochar is known to greatly increase the stable SOC fraction in amended soil [31,32,33] and the abundance and activity of soil microbiota as well as increase crop yields [34,35]. Moreover, biochar improves soil fertility by reducing nutrient leaching and mobility [36,37], thus providing more nutrients for plant utilization. However, such co-application of digestate and biochar was evaluated with different results [27,28,29,30]. Several other studies have shown adsorption of ammonium to biochar particles (up to 60%) to be associated with subsequent reductions in leaching [29], N_2_O- reduced atmospheric emissions [27,28], and positive effects on yields [30]. Changes, transformation dynamics, and the time-effect of these impacts on soil nutrient content have rarely been assessed. However, there are other materials used as soil amendments that can alter the properties of the digestate. Using elemental sulphur (S^0^) in combination with digestate could both increase the availability of sulphur, the lack of which leads to reduce in yields and quality of crops worldwide [38], and enhance the activity of soil microbes [39,40,41]. Plants usually take up sulphur in the form of sulphates while S^0^ is unavailable [42]. Since S^0^ activation (i.e., oxidation to sulphate SO_4_^2-^) is primarily a microbial process [43], its transformation in soil is stimulated when the soil microbial abundance and activity increases in general, e.g., due to the biochar application [35]. The slow and long-lasting impact of biochar and S^0^ application may determine conditions under which their positive effect on stability, reduced leaching, and gradually improved transformation of nutrients derived by digestate would be prolonged and still efficient even after repeated application of a fertilization dose of digestate. If digestate produced from food waste [44] from catering facilities, restaurants, kitchens, etc., is used, it would bring benefits in line with the circular economy approach.

Such a novel approach can contribute to the definition of a new organic fertilization procedure with enhanced efficacy and contribute to more sustainable agriculture.

Taking the above background into account, we intended to evaluate how mixing digestate with another type of amendment (biochar or elemental sulphur) would change the stability and microbial transformation of nutrients in soil amended with such a combined fertilizer and whether the resulting changes in microbial activity, nutrient availability, and their impact of maize biomass yield would be temporary or long-lasting. We hypothesized that:

i. The digestate enrichment with elemental sulphur or/and biochar enhances nutrient transformation (organic carbon and elemental sulphur oxidation) gradually. Therefore, joint significant pH changes, plant biomass increase, and higher indicators of microbial activity (enzymes) in the soil are comparable or more markable in themain cultivation (compared to the pre-cultivation).
ii. The biochar enhances the effect of the sulphur amendment and, when applied exclusively, could reduce soil acidity or retard nitrification (and reduce urease activity) over the long term.

## 2. Materials and Methods

### 2.1. Procurement of Materials for Preparation of Experimental Digestate and Pot Experiment

Digestate produced in a continuous mesophilic (≈40 °C) biogas plant (Czech Republic) processing food waste from catering facilities, restaurants, kitchens, etc. (properties in Table 1) was mixed with the amendments according to the doses mentioned in (Table 2) in 50 L barrels, which were then tightly sealed, and the mixture incubated at room temperature for six weeks. Commercially manufactured biochar was produced by pyrolysis of agricultural wastes (cereal bran and chaff, sunflower hulls, fruit peels, and pulp) at approx. 600 °C (Sonnenerde GmbH, Austria; properties are specified in Table 1), and waste sulphur was procured from the THIOPAQ biogas desulphurisation facility (Paques, The Netherlands).

After six weeks, the matured digestate was analysed for its properties, of which the values are shown in the Section 3 (Table 3): DM was determined gravimetrically (after drying at 105 °C to constant weight) on analytical scales. Mineral, ammonium, and nitrate nitrogen were measured according to [45]. Total sulphur content was determined according to [46]. For amoA, dsr, nirS, and 16S rDNA determination, DNA was extracted from 0.5 g of freeze-dried soil sample using the E.Z.N.A.^®^ Soil DNA Kit (Omega Bio-tek, Norcross, GA, USA). Isolated DNA was quantified using Nanodrop One (Thermo Scientific, Waltham, MA, USA). The SYBR-Green platform of Real-Time qPCR was used on a CFX96 Real-Time PCR detection system (Bio-Rad Laboratories, Hercules, CA, USA). Bacterial gene coding for amoA (ammonium monooxygenase) were multiplied using oligonucleotide primers amoA-1F (5′ GGGGTTTCTACTGGTGGT 3′) and amoA-2R (5′ CCCCTCKGSAAAGCCTTCTTC 3′) [47]; dsr was amplified with RH1-dsr-F (5′ GCCGTTACTGTGACCAGCC 3′) and RH3-dsr-R (5′ GGTGGAGCCGTGCATGTT 3′) [48]; nirS was amplified with nirSCd3aF (5‘ AACGYSAAGGARACSGG 3′) and nirSR3cd (5′ GASTTCGGRTGSGTCTTSAYGAA 3′) [49]; and 16S rDNA was amplified with 1108F (5′ ATGGYTGTCGTCAGCTCGTG 3′) and 1132R (5′ GGGTTGCGCTCGTTGC 3′) [50].

The matured digestate types were subsequently used as a soil amendment in a pot experiment with maize (*Zea mays* L.) to confirm its beneficial effects on crop growth and development. For this purpose, the pots having 2-L volume were filled with 1.7 kg of soil–sand mixture consisting of silty clay loam (USDA Textural Triangle) Haplic Luvisol, sieved through a 2-mm sieve and fine quartz sand (0.1–1.0 mm; ≥95% SiO_2_) mixed in a 1:1 weight ratio. A digestate in volume of 85 mL, equal to 50 m^3^·ha^−1^ (the dosage applied previously [51]), was applied to the soil surface and covered with an additional 0.3 kg of soil–sand mixture in order to simulate the application by injector below the soil surface, which reduces the release of ammonia emissions. Each pot was sown with 6 seeds of maize and placed in a greenhouse, and the experiment was carried out under seminatural conditions with all conditions controlled except light radiation. The temperature (day/night) was set to 20/12 °C, and the soil moisture was maintained at 65% of water holding capacity. The 12 h photoperiod with light intensity 370 µmol·m^−2^·s^−1^ was insignificantly affected by the solar radiation in the first part of the experiment (maize pre-cultivation = PreCult), which was carried out in winter. Later, the solar radiation increased the total light intensity in the second part of the experiment (the main cultivation = MCult) in spring, when the artificial light conditions (as well as the temperature and soil moisture conditions) were set the same as above (in PreCult).

After germination in PreCult, the seedlings were reduced to two (the most robust in each pot). The maize was grown for 12 weeks (December–February). Then the aboveground biomass (AGB) was cut at ground level, dried to constant weight at 60 °C, and weighed on laboratory scales. A mixed soil sample (100 g) was then taken from each pot by probe for further analysis. After sampling, each pot with the remaining soil and maize roots was fertilized with an additional dose of 70 mL (equal to 40 m^3^·ha^−1^) of control (unamended) digestate applied into 3 cm deep grooves, and the surface layer of the soil was lightly cultivated. Six maize (*Zea mays* L.) seeds were sown again. The same experimental conditions as used for the PreCult were applied for the MCult, lasting 12 weeks (March–May). A mixed soil sample (100 g) was then taken from each pot for further analysis.

### 2.2. Determination of Soil and Plant Properties

The soil samples were homogenized by sieving through a 2 mm sieve. Air-dried samples were used for determination of soil pH in CaCl_2_ [52], total soil carbon (TC), nitrogen (TN), and total sulphur (S) content using the Vario Macro Cube (Elementar Analysensysteme GmbH, Langenselbold, Germany). The freeze-dried samples were prepared for enzyme activity analyses: phosphatase (Phos), N-acetyl-β-D-glucosaminidase (NAG), arylsulfatase (ARS), and urease (Ure) [53].

The maize shoots were cut at ground level, and the roots were gently cleaned from the soil and washed with water. They were weighed on the analytical scales to determine the fresh aboveground biomass. The weighed shoots were dried at 60 °C to the constant weight, and dry AGB biomass was estimated gravimetrically by weighing the dried shoots on the analytical scales.

### 2.3. Statistical Analyses

The data obtained from the performed assays were statistically analysed using principal component analysis (PCA), one-way analysis of variance (ANOVA), the Tukey HSD post hoc test (at significance level *p* = 0.05), and Pearson correlation analysis (Program R, version 3.6.1) [54]. The results of the Pearson’s correlation analysis were mentioned when the value of the correlation coefficient r was: 0.5 < r < 0.7 (moderate correlation) and 0.7 < r < 0.9 (high correlation) [55].

## 3. Results

### 3.1. Aboveground Biomass Yield, Soil Nitrogen and Sulphur Content

**Figure 1:**
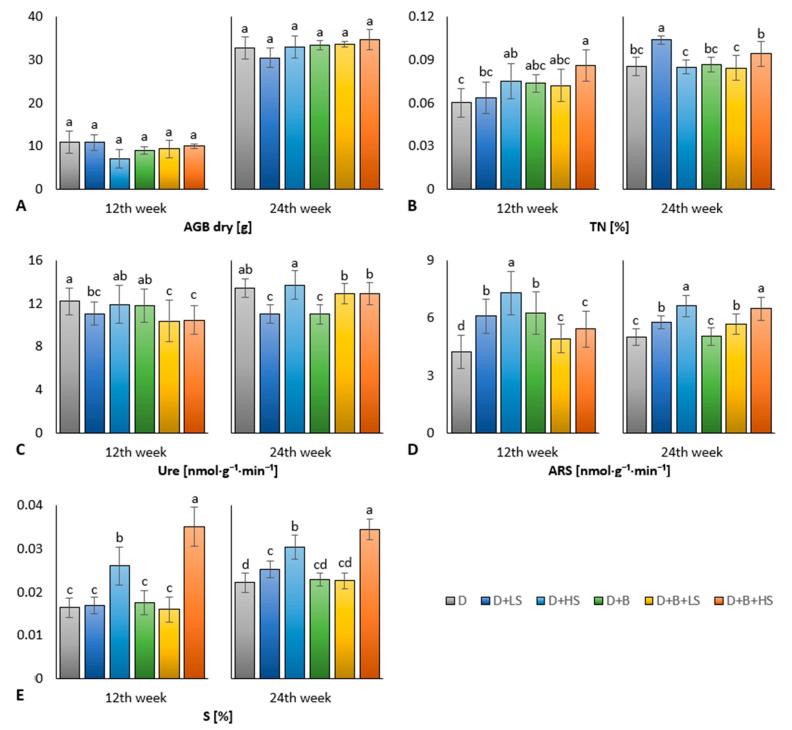
Dry aboveground biomass (**A**), soil total nitrogen (**B**), urease (**C**), and arylsulfatase activity (**D**), total sulphur (**E**) of experimental variants in the pre-cultivation (12th week) and the main cultivation (24th week) of maize in the pot experiment. Average values are displayed, error bars are standard deviation. The different letters of variant values indicate a statistical difference between them at the level *p* ≤ 0.05.

In both parts of the experiment, the maize AGB dry showed no statistically significant differences among the variants (Figure 1A). However, there was a difference in AGB dry values in the PreCult and the MCult. The higher AGB values in the MCult might have been caused by higher contribution of natural light in spring compared to winter, or by a higher growth-promoting effect of the residual digestate from PreCult plus additional digestate added in MCult and decomposed residues of previously grown maize roots. The positive effect of D + B + HS amendment on nitrogen sequestration was reflected by the significantly highest total nitrogen (TN) content in the PreCult. The MCult revealed significantly highest TN for the D + LS variant, followed by the D + B + HS variant. We expected that the highest TN content in the D + LS variant in the MCult may have been a predictor for increased urease (Ure) and N-acetyl-glucosaminidase (NAG) activities and enhanced nitrogen mineralization rate in the soil. However, we observed the opposite results: variants D + LS and D + B resulted in the significantly lowest urease (Ure) compared to the control (D) in MCult (Figure 1C). Similarly, the variants D + B + LS and D + B + HS showed a significantly reduced Ure activity compared to the other variants in the PreCult.

In the PreCult, arylsulfatase (ARS) significantly increased in all the amended variants. The significantly highest ARS value was detected in D + HS, followed by D + LS and D + B (Figure 1D). In the MCult, the D and D + B variants exerted the significantly lowest ARS, whereas the D + HS and D + B + HS had the significantly highest ARS (Figure 1D).

Digestate enrichment with high S^0^ had the strongest effect on the soil sulphur content in the PreCult, which was reflected by significantly increased S values in the D + HS and D + B + HS variants, (Figure 1E). Moreover, the significantly highest S content was found in the variant D + B + HS in the MCult, and the D + HS and D + LS showed significantly increased S content compared to the control (D) (Figure 1E).

### 3.2. Enzymes, Soil Carbon Content, and pH

**Figure 2:**
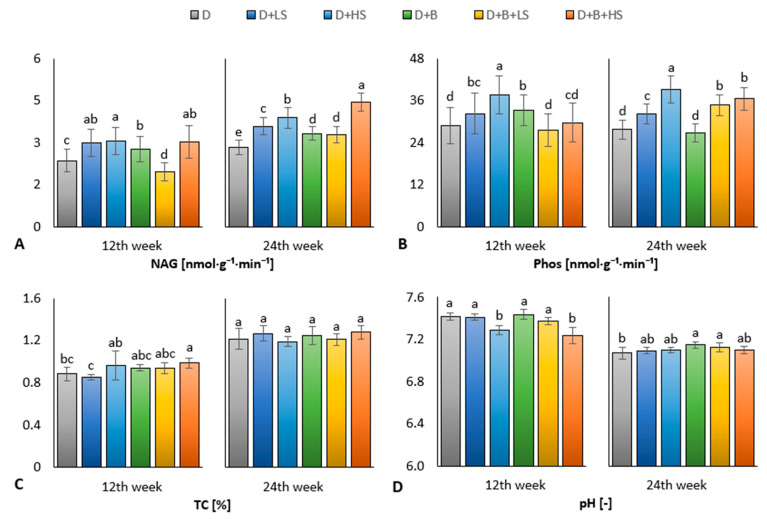
Soil activity of N-acetyl-β-D-glucosaminidase (**A**), phosphatase (**B**), soil total carbon (**C**), and soil pH (**D**) of experimental variants in PreCult (12th week) and MCult (24th week) of maize in the pot experiment. Average values are displayed, and error bars are standard deviation. The different letters of variant values indicate a statistical difference between them at the level *p* ≤ 0.05.

Sulphur-enriched digestate positively impacted the activity of NAG. In the PreCult, all the variants (except the D + B + LS) showed significantly increased NAG compared to the control (D). The highest value was found in the D + HS variant followed by the D + LS and D + B + HS, Figure 2A. A positive effect of sulphur addition (to digestate) on the NAG activity was also found in the MCult. The highest value was found in the D + B + HS variant followed by the D + HS and D + LS (Figure 2A).

Moreover, enrichment of digestate with sulphur positively affected the activity of phosphatase (Phos) in the amended soil, mainly in the MCult. The variant D + HS showed the highest Phos in both parts of the experiment (Figure 2B). In addition, this was followed by variants D + B + LS, D + B + HS and D + LS at the end of the experiment.

In the PreCult, a significantly increased total carbon (TC) was found in the D + B + HS variant compared to the control (D) and D + LS (Figure 2C). In contrast, TC showed no significant difference among the variants in the MCult (Figure 2C). Significantly decreased pH was found in variants D + HS and D + B + HS in the PreCult (Figure 2D). At the end of experiment, the significant increase in pH value was detected only in variants D + B and D + B + LS (Figure 2D).

## 4. Discussion

### 4.1. Aboveground Biomass Yield, Soil Nitrogen and Sulphur Content

Insignificant differences between the maize AGB dry values of all six variants in both phases of the experiment (Figure 1A) indicated that various digestate types had neither a beneficial nor an adverse effect on the maize biomass (Figure 1A). However, there are also studies which revealed a positive effect of elemental sulphur on crop nutrition, growth, and yield [42,56,57].

**Figure A1:**
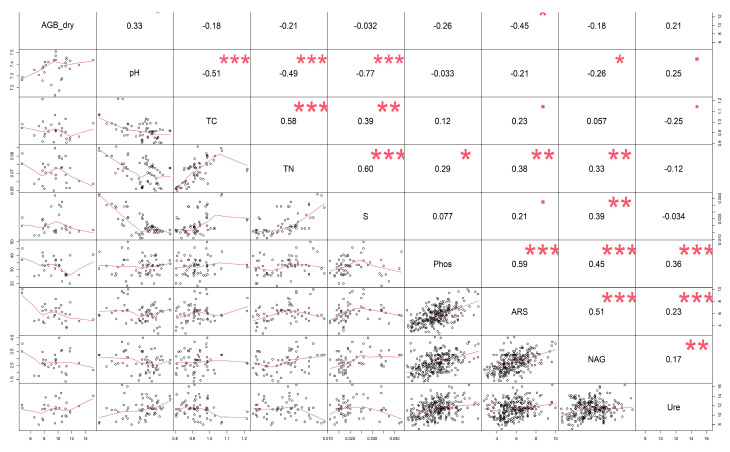
Results of Pearson’s correlation analysis in the pre-cultivation of maize in the pot experiment. The symbols indicate differences between the variables at the following levels of statistical significance: ˙ *p* ≤ 0.1, * *p* ≤ 0.05, ** *p* ≤ 0.01, *** *p* ≤ 0.001.

The highest TN for D + B + HS in first part of the experiment can be explained by biochar-mediated adsorption of ammonium in the amended soil [58,59], as ammonium is the predominant form of nitrogen in the digestate. The highest TN for D + LS in the second part might have also been coupled with putatively reduced nitrogen mineralization (which prevented nitrogen losses, e.g., via volatilization or leaching); this is indicated by decreased urease activity (Figure 1C) concurrently leading to reduced ammonia emissions due to the effect of the S^0^ in the respective digestate variant. Reduced ammonia volatilization due to the application of elemental sulphur to the soil has also been reported in previous studies [60], because S^0^ contributes to nitrogen immobilization by increased content of the NH_4_^+^ form. The digestate enriched with low S^0^ and the digestate enriched with biochar in the amended soil sequesters nitrogen sources in the long term through stabilization and mitigation of nitrification. Thus, the higher nitrogen content of the digestate-enriched soil was probably due to a decrease in Ure activity, which was probably derived from the higher access of the sulphur as reported by Gupta et al. (1988) [61] and its increased transformation (measured as ARS arylsulfatase activity) in the first part of the experiment. This assumption was confirmed by a significant positive correlation of TN with S (r = 0.6, *p* ≤ 0.001) (Figure A1). An inhibitory effect of an intermediate conversion of a sulphur such as thiosulfate can probably be considered (similarly as referred [62]), because the natural oxidation of elemental sulphur via thiosulfate to sulphates occurs in soils [63,64].

The primary increase in ARS activity by digestate was observed for all the variants in the PreCult. However, only the D + HS variant showed significantly higher ARS compared to the value of D + LS digestate treatment in the PreCult. In the MCult, the ARS fully reflected the dose of added S^0^ that was applied to the soil together with the treated digestate. The ARS was most elevated in the D + HS and D + B + HS treatments, with the control (D) and D + B showing the lowest values. The positive effect of the S^0^-enriched digestate on the sulphur mineralization activity in the soil was confirmed by a significant positive correlation between ARS and S (*p* ≤ 0.001, r = 0.63) in the MCult. Nevertheless, both digestate variants combining biochar and S^0^ showed weaker stimulation of ARS (compared to D + B variant) in the PreCult; this can be explained by a possible retardation of S^0^ utilization in the soil due to a stabilizing interaction with the co-amended biochar. Subsequently, in a significant positive effect, S^0^ + biochar was lagged and finally manifested as an ARS stimulation by D + B + LS and D + B + HS in the MCult.

**Figure A2:**
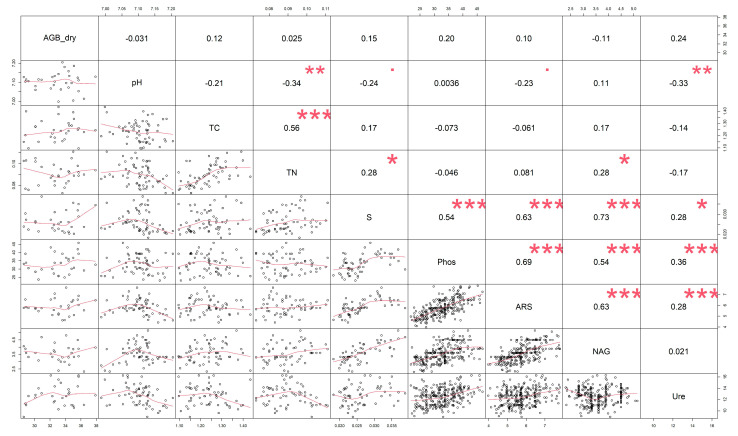
Results of Pearson’s correlation analysis in the main cultivation of maize in the pot experiment. The symbols indicate differences between the variables at the following levels of statistical significance: ˙ *p* ≤ 0.1, * *p* ≤ 0.05, ** *p* ≤ 0.01, *** *p* ≤ 0.001.

During the whole experiment, the soil enriched with both digestates with higher S^0^ content showed the highest S content (Figure 1E). However, variant D + B + HS contained significantly more S in both parts of the experiment compared to D + HS, which may be attributed to the addition of biochar to the digestate. The findings showed that biochar enhances sulphur adsorption in amended soil [65,66]. The Pearson correlation analysis revealed a significant (*p* ≤ 0.001) positive (r = 0.6) correlation of S with TN and a negative correlation with pH (r = −0.77) in the PreCult, indicating that digestate enrichment with sulphur mediated a joint effect on nitrogen sequestration (retardation of nitrification) and acidification of the treated soil. The long-term effect (part 2 of the experiment) of elevated soil sulphur was associated with an increase in soil enzyme activity, as confirmed by significant correlations (*p* ≤ 0.001) of S with Phos (r = 0.54), NAG (r = 0.73), ARS (Figure A2).

### 4.2. Enzymes, Soil Carbon Content, and pH

Specifically, there was an increase in NAG activity, which was highest in the D + HS variant in the PreCult of the experiment and second highest in the D + B + HS variant, which was also significantly highest at the end of the experiment. The NAG has been recognized as related to the fungal abundance and activity in soil [67]. The findings show that increased NAG content indicates higher fungal biomass and its turnover in the MCult, which was demonstrated by the significant correlation (*p* ≤ 0.001) between NAG and Phos (r = 0.54). The NAG was correlated (*p* ≤ 0.001) with Phos (r = 0.45) also in the first phase of the experiment, which was explained by a synergistic relationship between nitrogen mineralization by the arbuscular mycorrhizal fungi (AMF) and phosphate solubilization [68,69]. AMF growth stimulation could be mediated by the addition of S^0^-enriched digestate to the arable soil, similar to the finding that sulphur-rich areas were found to correlate with higher hyphal density and increased organically bound P-pool in the plant soil–AMF interaction model [70].

This predicted benefit of S^0^-enriched digestate for Phos activity in amended soil was particularly evident in the D + HS variant, which showed the highest Phos values both in part 1 and at the end of the experiment. Moreover, Phos values significantly increased in the variants D + B + LS and D + B + HS in the end but not in the PreCult. Soluble phosphate in digestate has been reported to improve nutrient use efficiency in amended soils [71]; however, reduced phosphatase activity in soils fertilized with biochar and digestate compared to the control unenriched digestate has already been observed [51] and is similar to the negative primary effect on Phos in this study.

Despite the apparent effects of the addition of biochar to the digestate in both phases of the experiment, the TC did not change significantly in the MCult (Figure 2C). In contrast, the total carbon content increased in the D + B + HS variant compared to the control (D) and D + LS (Figure 2C) in the PreCult. The D + HS variant also showed high TC value, in comparison to D + LS, which significantly increased (Figure 2C). The effect of a high S^0^-enriched digestate on total carbon content is probably comparable to that of a biochar-enriched digestate. However, the association of sulphur transformation with carbon sequestration has only been observed in the marine environment [72,73]. The beneficial effect of digestate enriched with biochar and S^0^ on the TC content of treated soil has also been observed in our previous study [51]. Other studies investigated the relationship between carbon and sulphur mineralization [74,75] and the dependence of the mineralization rate of carbon sources on sulphur addition to the soil [76,77]. In line with these studies, we presumed that higher TC prerequisites increased carbon mineralization and predicted higher microbial carbon uptake in the D + HS and D + B + HS variants, which is attributed to the observed higher TN and S contents and NAG activity and the lower pH (approaching neutral) in the variants supplemented with high S^0^-enriched digestate. This observation is consistent with the long-term negligible effect of biochar on TC and can be explained by the increase in carbon conversion by sulphur; but the different ratios between labile and recalcitrant carbon in the different variants must be considered.

All the observed differences can be attributed to differences in pH in the PreCult; as expected, the D + HS and D + B + HS variants were the most acidified due to the high dose of S^0^ in the treated digestate, Figure 2D. This acidifying effect of a large dose of S^0^ added to the soil was mainly observed with one-time application [40,78]. However, this effect was weakened at the end of the experiment, when the pH of all the soil variants was reduced. The alkalizing effect of biochar was evident in the D + B and D + B + LS variants with significantly increased pH compared to the control (Figure 2D). This means that biochar addition to low elemental sulphur S counteracted its acidifying potential. The impact of digestate enriched with biochar or biochar + a low dose of S^0^ on pH changes was in agreement with the findings of other authors [79,80].

## 5. Conclusions

The aim of this pot experiment was to compare the short- and long-time effect of fertilization with a digestate enriched with biochar, elemental sulphur in two doses, and a combination of both elements on soil properties and dry biomass of maize. As hypothesized, the revealed differences were conditioned by the various amendments and by the soil amendment interaction intervals. We concluded that the most significant differences were mediated by application of a digestate enriched with a high dose of elemental sulphur. Total nitrogen, carbon, and sulphur increased under the short-term experiment, while sulphur content, arylsulfatase activity, N-acetyl-β-D-glucosaminidase, phosphatase, and non-inhibited urease of the digestate enriched with high elemental sulphur and biochar increased under the long-term experiment. The hypothesized negative priming effect of biochar on urease, arylsulfatase, and phosphatase activity was proven. On the other hand, elemental sulphur enhanced N-acetyl-β-D-glucosaminidase, arylsulfatase, and phosphatase activities in the second phase of experiment. We verified the hypothesized prolonged S^0^-mediated increase in nutrient transformation (carbon mineralization), which led to comparable total carbon content in the MCult. The acidifying effect of a high dose of S^0^ (alone or with biochar) in the PreCult was mitigated in the MCult. We did not observe any significant effect of applied amendments on the dry aboveground biomass of the maize. Such variable results suggest a need for further research exploring the residual and prolonged effects of applied amendments under varying experimental conditions. Future steps in the progress of this research topic will include more experimentation under diverse conditions and with other plant species.

## Figures and Tables

**Table 1:** Properties of soil amendments.

Property [Unit]	Digestate ^(1)^	Biochar ^(2)^
DM [%]	6.0 ± 0.1	65–75
BET [m^2^·g^−1^]	-	289
Ash_550°C_ [%]	not measured	11.7
C_tot_ [g·kg^−1^]	not measured	866
C_org_ [g·kg^−1^]	not measured	74
N_tot_ [g·kg^−1^]	43.8 ± 3.39	13.2
P [g·kg^−1^]	0.8 ± 0.05	6.2
S [g·kg^−1^]	0.8 ± 0.06	not measured
Ca [g·kg^−1^]	1.2 ± 0.1	8.1
Mg [g·kg^−1^]	0.1 ± 0.01	6.7
K [g·kg^−1^]	1.4 ± 0.13	24.4

Displayed are average values of properties and nutrient content in: (1) fresh matter, n = 3, ±standard deviation (SD), (2) dry matter: DM = dry matter; BET = Brunauer–Emmett–Teller surface area; Ash_550°C_ = ash content at ignition temperature 550 °C; C_tot_ = total carbon content; C_org_ = organic carbon content; N_tot_ = total nitrogen content; P = total phosphate; S = total sulphur; Ca, Mg, K = total content of respective elements.

**Table 2:** Experimental variants of digestate.

Variant	Abbrev.	Digestate per Barrel [L]	Biochar per Barrel [g]	Sulphur per Barrel [g]
Digestate	D	10	-	-
Digestate + low dose of sulphur	D + LS	10	-	14
Digestate + high dose of sulphur	D + HS	10	-	140
Digestate + biochar	D + B	10	400	-
Digestate + biochar + low dose of sulphur	D + B + LS	10	400	14
Digestate + biochar + high dose of sulphur	D + B + HS	10	400	140

**Table 3:** Properties of matured digestate expressed in fresh matter.

variant	DM	N_min_	N-NO_3_	N-NH_4_	S
	[%]	[g∙kg^−1^]			
D	5.96 ± 0.39 c	7.37 ± 0.45 c	2.20 ± 0.13 c	5.17 ± 0.32 b	0.85 ± 0.04 d
D + LS	6.86 ± 0.51 b	8.96 ± 0.51 a	4.38 ± 0.31 a	4.58 ± 0.28 c	0.64 ± 0.05 e
D + HS	5.78 ± 0.32 c	7.09 ± 0.40 c	1.64 ± 0.11 d	5.45 ± 0.28 b	0.60 ± 0.03 e
D + B	6.75 ± 0.40 b	8.26 ± 0.504 b	2.16 ± 0.16 c	6.10 ± 0.38 a	1.94 ± 0.11 b
D + B + LS	6.67 ± 0.41 b	5.32 ± 0.31 d	1.76 ± 0.12 d	3.56 ± 0.26 d	1.52 ± 0.10 c
D + B + HS	8.06 ± 0.57 a	9.14 ± 0.48 a	3.07 ± 0.16 b	6.07 ± 0.43 a	2.39 ± 0.13 a
**variant**	**amoA**	**dsr**	**nirS**	**16S rDNA**	
	**[cps∙g^−1^]**				
D	3.81∙10^5^ ± 2.45∙10^4^ c	0.89∙10^7^ ± 0.41∙10^6^ e	3.15∙10^6^ ± 1.73∙10^5^ b	0.57∙10^11^ ± 0.42∙10^10^ d	
D + LS	1.16∙10^5^ ± 0.57∙10^4^ e	2.56∙10^7^ ± 1.61∙10^6^ c	0.59∙10^6^ ± 0.36∙10^5^ e	0.22∙10^11^ ± 0.14∙10^10^ e	
D + HS	5.67∙10^5^ ± 4.02∙10^4^ a	5.88∙10^7^ ± 4.48∙10^6^ a	3.01∙10^6^ ± 2.18∙10^5^ b	1.73∙10^11^ ± 0.86∙10^10^ a	
D + B	4.89∙10^5^ ± 3.65∙10^4^ b	2.97∙10^7^ ± 1.64∙10^6^ b	3.72∙10^6^ ± 1.95∙10^5^ a	1.42∙10^11^ ± 1.00∙10^10^ b	
D + B + LS	4.46∙10^5^ ± 2.55∙10^4^ b	1.64∙10^7^ ± 1.24∙10^6^ d	2.40∙10^6^ ± 1.49∙10^5^ c	1.13∙10^11^ ± 0.52∙10^10^ c	
D + B + HS	3.12∙10^5^ ± 1.98∙10^4^ d	1.66∙10^7^ ± 1.00∙10^6^ d	1.82∙10^6^ ± 0.93∙10^5^ d	0.61∙10^11^ ± 0.45∙10^10^ d	

DM = dry matter; N_min_ = mineral nitrogen; N-NO_3_ = nitrate nitrogen; N-NH_4_ = ammonium nitrogen; S = total sulphur; amoA = soil gene copy number, indicator of nitrifying microorganisms; dsr = soil gene copy number, indicator of sulphur-reducing microorganisms; nirS = soil gene copy number, indicator of denitrifying microorganisms; 16S (rDNA) = gene copy number, indicator of bacteria in digestate. Average values ± standard deviation are displayed. The different letters of variant values indicate a statistical difference between them at the level *p* ≤ 0.05.

## Data Availability

The data presented in this study are available on request from the corresponding author.
